# P-1505. Cellular and Humoral Immunogenicity of 1 or 2 injections of mRNA-1403, a Multivalent Norovirus mRNA Vaccine, in Healthy Adults

**DOI:** 10.1093/ofid/ofaf695.1689

**Published:** 2026-01-11

**Authors:** Rekha R Rapaka, Meklit Workneh, Brooke A Bollman, Alexander Rumyantsev, Till Schoofs, Jaap Oostendorp, Gabrielle Fortier, Kevin Mancini, Wenlin Yuan, Lauren Bailey, Tin Bartholomew, Shannon McGrath, Heather L Cahill, Agi Buchanan, Katherine B Carlson, Stephen J Schrantz, Doran Fink

**Affiliations:** Moderna Therapeutics, Cambridge, Massachusetts; Moderna, Inc, Cambridge, Massachusetts; Moderna, Inc, Cambridge, Massachusetts; Moderna, Inc, Cambridge, Massachusetts; Moderna, Inc, Cambridge, Massachusetts; Moderna, Inc, Cambridge, Massachusetts; Moderna, Salt Lake City, Utah; Moderna, Inc, Cambridge, Massachusetts; Moderna, Inc, Cambridge, Massachusetts; Moderna, Inc, Cambridge, Massachusetts; Moderna, Inc, Cambridge, Massachusetts; Moderna, Inc., Norwood, Massachusetts; Moderna Tx, Norwood, Massachusetts; Moderna, Inc., Norwood, Massachusetts; Moderna, Salt Lake City, Utah; Moderna, Salt Lake City, Utah; Moderna, Inc, Cambridge, Massachusetts

## Abstract

**Background:**

Norovirus (NoV) is a non-enveloped, positive-sense RNA virus in the family Caliciviridae and is a leading cause of acute gastroenteritis (AGE) worldwide. There are currently no approved vaccines for prevention of NoV AGE, and due to the broad and shifting diversity of circulating NoVs, a multivalent approach is likely needed to achieve sufficient breadth for protection. mRNA-1403 is a trivalent mRNA-based NoV vaccine candidate consisting of mRNAs encoding for the major capsid protein (VP1) of 3 globally prevalent NoV genotypes formulated in a lipid nanoparticle.

**Methods:**

This ongoing Phase 1/2, randomized, placebo-controlled, observer-blind, dose-ranging study in healthy adults 18-80 years of age (NCT05992935) evaluated the safety and immunogenicity of mRNA-1403 at up to 4 dose levels and administered as 1 or 2 injections. Genotype-specific anti-VP1 memory T cell responses, functional antibody responses (HBGA blocking antibody responses), and binding antibody responses were assessed in older and younger adults, at baseline and at 28 days after each injection. NoV specific CD4+ and CD8+ T cell memory populations (T_EM_, T_EMRA_, and T_CM_) were characterized in PBMCs stimulated with VP1 peptide pools and assessed for expression of surface and intracellular effector function markers by flow cytometry.

**Results:**

Vaccination with mRNA-1403 elicited VP1 genotype specific polyfunctional CD4+ and CD8+ T cell responses against all three NoV genotypes included in the vaccine. A general dose response effect was observed with stronger responses seen at higher doses. The CD4+ T cell response elicited upon vaccination is Th1 predominant. Polyfunctional memory T cell responses were of similar quality in older and younger adults. Additionally, mRNA-1403 elicited a strong HBGA blocking antibody response against all genotypes included in the vaccine. Antibody and T cell responses trended higher by 28 days after the second injection of mRNA-1403, most notably against GII.4 VP1.

**Conclusion:**

mRNA-1403 elicits robust, highly polyfunctional genotype-specific CD4+ and CD8+ memory T cells as well as genotype-specific anti-VP1 blocking and binding antibodies in adults.

**Disclosures:**

Rekha R. Rapaka, MD, PhD, Moderna Therapeutics: Employee at Moderna Therapeutics|Moderna Therapeutics: Stocks/Bonds (Private Company) Meklit Workneh, MD, MPH, Moderna: Stocks/Bonds (Public Company) Brooke A. Bollman, PhD, Moderna: Currently employed by Moderna|Moderna: Stocks/Bonds (Public Company) Alexander Rumyantsev, MD, PhD, Moderna: Stocks/Bonds (Public Company) Till Schoofs, MD, GlaxoSmithKline Biologicals: Employee|Moderna, Inc: Stocks/Bonds (Public Company) Jaap Oostendorp, PhD, Moderna, Inc.: Employee|Moderna, Inc.: Stocks/Bonds (Public Company) Gabrielle Fortier, MPH, Moderna: Stocks/Bonds (Public Company) Kevin Mancini, MS, Moderna: Employee|Moderna: Stocks/Bonds (Public Company) Wenlin Yuan, MS, PhD, Moderna: Stocks/Bonds (Private Company) Lauren Bailey, PhD, Moderna Inc: Stocks/Bonds (Public Company) Shannon McGrath, MS, Moderna, Inc.: Employee|Moderna, Inc.: Stocks/Bonds (Public Company) Heather L. Cahill, BSc, Moderna Tx: Stocks/Bonds (Public Company) Agi Buchanan, MD, PhD, Moderna, Inc.: Stocks/Bonds (Public Company) Katherine B. Carlson, PhD, MPH, Moderna: Employee|Moderna: Stocks/Bonds (Private Company) Stephen J. Schrantz, Jr., MD, Moderna: Stocks/Bonds (Public Company) Doran Fink, MD, PhD, ModernaTX, Inc.: Employee|ModernaTX, Inc.: Stocks/Bonds (Public Company)

